# Disrupted Human–Dog Interbrain Neural Coupling in Autism‐Associated *Shank3* Mutant Dogs

**DOI:** 10.1002/advs.202402493

**Published:** 2024-09-11

**Authors:** Wei Ren, Shan Yu, Kun Guo, Chunming Lu, Yong Q. Zhang

**Affiliations:** ^1^ State Key Laboratory for Molecular Developmental Biology Institute of Genetics and Developmental Biology Chinese Academy of Sciences Beijing 100101 China; ^2^ College of Life Sciences University of Chinese Academy of Sciences Beijing 100049 China; ^3^ Laboratory of Brain Atlas and Brain‐inspired Intelligence Institute of Automation, Chinese Academy of Sciences Beijing 100190 China; ^4^ School of Psychology University of Lincoln Brayford Pool Lincoln LN6 7TS UK; ^5^ State Key Laboratory of Cognitive Neuroscience and Learning & IDG/McGovern Institute for Brain Research Beijing Normal University Beijing 100875 China; ^6^ School of Life Sciences Hubei University Wuhan 430062 China

**Keywords:** autism spectrum disorders, human–dog dyads, interbrain neural couplings, lysergic acid diethylamide, *Shank3*

## Abstract

Dogs interact with humans effectively and intimately. However, the neural underpinnings for such interspecies social communication are not understood. It is known that interbrain activity coupling, i.e., the synchronization of neural activity between individuals, represents the neural basis of social interactions. Here, previously unknown cross‐species interbrain activity coupling in interacting human–dog dyads is reported. By analyzing electroencephalography signals from both dogs and humans, it is found that mutual gaze and petting induce interbrain synchronization in the frontal and parietal regions of the human–dog dyads, respectively. The strength of the synchronization increases with growing familiarity of the human–dog dyad over five days, and the information flow analysis suggests that the human is the leader while the dog is the follower during human–dog interactions. Furthermore, dogs with *Shank3* mutations, which represent a promising complementary animal model of autism spectrum disorders (ASD), show a loss of interbrain coupling and reduced attention during human–dog interactions. Such abnormalities are rescued by the psychedelic lysergic acid diethylamide (LSD). The results reveal previously unknown interbrain synchronizations within an interacting human–dog dyad which may underlie the interspecies communication, and suggest a potential of LSD for the amelioration of social impairment in patients with ASD.

## Graphical abstract

This study explores interbrain activity coupling during human–dog interactions, revealing that mutual gaze and petting enhance neural synchronization in frontal and parietal regions, respectively. Dogs carrying autism‐linked *Shank3* mutations display impaired neural coupling and attention, which are rescued by the psychedelic lysergic acid diethylamide. These findings offer insights into interspecies communication and potential autism therapies.

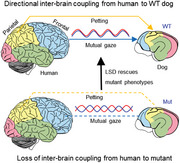

## 1 Introduction

The communication between humans and dogs has evolved over 30 000 years, with dogs being the first domesticated by humans for their hunting skills and protective abilities.^[^ ^1^ ^]^ Over time, dogs have become integral members of many families, providing emotional support and companionship. While some interspecies relationships are formed based on mutual benefits such as protection, they rarely achieve the same level of communication seen in human–dog pairs. Moreover, dogs have evolved to read, understand and respond to a wide range of human emotional states and communicative signals through behaviors, facial expressions, and even vocal tones,^[^ ^2^, ^3^, ^4^ ^]^ offering an extraordinary level of active companionship that is not often seen in other domesticated or companion animals, such as cats. However, the neural mechanisms underlying the distinctive and effective communication between humans and dogs are largely unknown.^[^ ^5^ ^]^

During social interactions, interacting individuals are not isolated, but are embedded in a multibrain system.^[^ ^6^, ^7^ ^]^ Previous studies have discovered that animals within the same species display interbrain neural coupling during social interactions. These neural couplings were initially observed in humans^[^ ^8^, ^9^ ^]^ and subsequently found in mice,^[^ ^10^ ^]^ bats,^[^ ^11^ ^]^ and nonhuman primates.^[^ ^12^ ^]^ Interbrain neural coupling was shown to reflect reciprocity in social interactions, joint attention, and the quality and outcome of social interactions.^[^ ^7^ ^]^

While previous studies on interbrain neural coupling have exclusively focused on interactions within a species, it is not yet known whether interbrain activity coupling also occurs between individuals of different species. The unique attachment between humans and dogs raises important questions of how the neural states of dogs and humans may couple with each another when they interact, how this may reflect their internal states of the ongoing social interactions such as joint attention, and how this may vary with dogs’ ability to interact with humans. The present study aims to investigate if the coupling exists, and if so, whether autism‐associated gene mutations in dogs can impair the social interaction between humans and dogs.

In this study, we utilized noninvasive wireless electroencephalogram (EEG) to simultaneously measure brain activity in laboratory dogs (beagles) and unfamiliar humans while they engaged in social interactions. We demonstrated for the first time that directed interbrain neural coupling occurs between humans and dogs, particularly in the frontal and parietal regions, both of which are associated with joint attention. Furthermore, we discovered that autism‐associated *Shank3* mutations in dogs abolished the interbrain neural coupling and joint attention during human–dog interactions, and these phenotypes were rescued by a single dose of the psychedelic lysergic acid diethylamide (LSD), which reopens the critical period of social reward learning in mice.^[^ ^13^ ^]^ Our findings have implications for understanding the neural mechanisms underlying the effective social interaction between family dogs and humans and suggest a potential of LSD in ameliorating the social deficits in ASD.

## 2 Results

### 2.1 Interbrain Neural Coupling between Humans and Dogs

**Figure 1:**
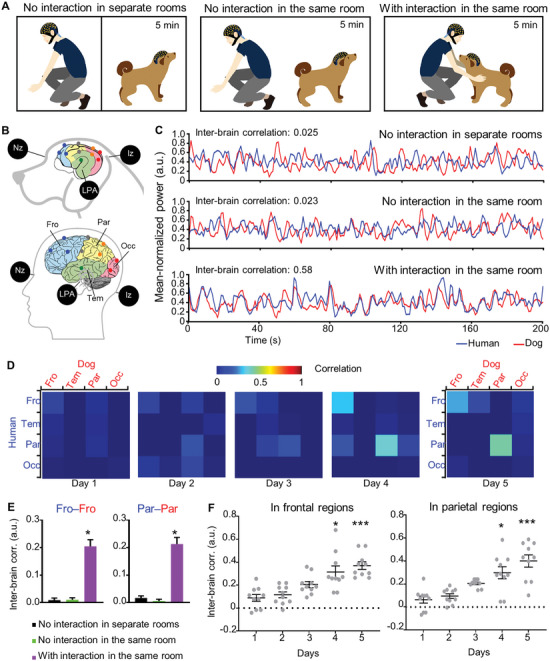
Interbrain activity coupling during human–dog interactions. A) Schematic of human–dog interactions (mutual gaze and petting): no interaction in separate rooms (left), with (right panel) and without (middle panel) interactions in the same room. B) Schematic of scalp electrode positions on dog brain (top) and human brain (bottom). The electrodes are color‐coded (blue: frontal region; orange: parietal region; green: temporal region red: occipital region). Nz: the bridge of the nose; LPA: the left ear canals; RPA: the right ear canals; Iz: the little bump at the very back of the skull. C) Mean normalized EEG powers simultaneously recorded in frontal regions of dog and human on the fifth day of social interactions in three different conditions (no social interaction in separate rooms (top), and in the same room (middle), and with social interactions in the same room (bottom)). D) Heat map of Interbrain activity correlations between socially interacting dogs and humans over five days. The color bar from blue to red indicates the correlation coefficient ranging from 0 to 1. Fro: frontal region; Tem: temporal region; Par: parietal region; Occ: occipital region. E) Interbrain correlation in frontal and parietal regions of dog (red) and human (blue) brain. *p < 0.05. *n* = 10. Error bars represent SEM. F) Interbrain correlation in frontal regions (left) and parietal regions (right) of socially interacting dog and human over five days. *p < 0.05, ***p < 0.001 compared with the first day, Friedman test.

To investigate brain activity dynamics in human–dog dyads during social interactions, we employed noninvasive wireless EEG to simultaneously record brain activity in dogs and humans. The scalp electrode positions are illustrated in **Figures** **1**B and S1 (Supporting Information), with 16 electrodes covering the frontal, parietal, temporal, and occipital cortices. In the experimental setup (Figure 1A), we recorded EEG data from a dog and a human participant simultaneously in three different conditions, namely, in separate rooms without social interactions, and with and without social interactions involving mutual gaze and petting in the same room. As EEG signals are heavily influenced by movements, we integrated wavelet transformation and neural networks^[^ ^14^, ^15^ ^]^ to detect and remove EEG artifacts. Our analysis of EEG data revealed strong neural activity correlations (Pearson correlation coefficients were used to quantify correlation strengths) in the dog and human brains on the fifth day of human–dog interactions (Figure 1C).

Figure 1D provides an example of the interbrain correlation in frontal and parietal regions, which increased as the human–dog dyads interacted repeatedly across five days. To validate our observation, we calculated the interbrain correlation between these brain regions in three conditions (no social interactions in separate rooms and with or without social interactions in the same room, Figure 1A), and found that interbrain correlation in the frontal and parietal regions of the human–dog dyads was higher than that in other brain regions during social interactions (Figure 1D,E).

It could be argued that the interbrain activity coupling reflects generic neural activity associated with specific components of social interactions (e.g., processing of comparable sensory inputs such as facial signals) irrespective of whether participants are directly interacting and that this effect may lead to false‐positive results. To rule out this possibility, we analyzed neural activity correlation across pairs of human–dog dyads, i.e., the neural activity of humans in one trial and that of dogs in another trial. Our analysis showed that interbrain correlations across pairs of human–dog dyads from different sessions were significantly lower than those from the same interacting sessions (*p* < 0.05, Mann‒Whitley *U* test, Figure S2, Supporting Information), confirming that mutual engagement between the dog and human is necessary for interbrain activity coupling.

During human–human interactions, once individuals become more familiar with each other, they will develop increased interbrain activity coupling between each other.^[^ ^9^, ^16^, ^17^ ^]^ To investigate whether social experience with each other affected interbrain activity coupling between dogs and humans, we recorded the brain activity of dogs and humans during social interactions across five days. The dog was unfamiliar with the experimenter before the experiment. We found that interbrain activity coupling significantly increased from 0.09 ± 0.03 and 0.06 ± 0.03 on the first day to 0.37 ± 0.03 and 0.40 ± 0.05 on the fifth day in frontal regions and parietal regions, respectively (Figure 1F). The linear regression results showed a significant positive relationship between the social interaction duration and interbrain activity correlation (β = 0.076, SEM = 0.011, *p* < 0.01 in the frontal regions; β = 0.088, SEM = 0.012, *p* < 0.01 in the parietal regions).

To see if the interbrain correlation persisted or changed over a longer period, we extended the analysis of interbrain correlation for five more days after the original 5‐d social interactions in three additional dogs. The results from logistic growth curve regression analysis showed that interbrain correlation in both frontal and parietal regions reached a plateau on the seventh day of social interactions (Figure S3, Supporting Information).

### 2.2 Mutual Gaze and Petting Induce Interbrain Activity Coupling in distinct Brain Regions

Mutual gaze and social touch are two fundamental forms of nonverbal communication.^[^ ^18^, ^19^ ^]^ During mutual gaze, specific human dyads in discussion spontaneously adopt leader/follower roles, resulting in an increased interbrain synchronization.^[^ ^20^, ^21^ ^]^ Social touch refers to any physical contact intended to communicate emotions, establish social bonds, or convey information.^[^ ^22^ ^]^ Petting refers to the act of stroking or caressing an animal, usually a pet, to show affection or provide comfort. Petting has positive effects on both the pet and the pet owner, including stress reduction, increased social interactions, and improved mood.^[^ ^23^ ^]^

**Figure 2:**
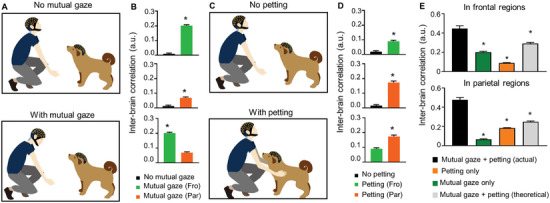
Mutual gaze and petting facilitate interbrain activity coupling in frontal regions and parietal regions, respectively. A) Schematic of human–dog interactions with (bottom) and without (top) mutual gaze. B) The interbrain activity couplings in frontal regions of dog and human increase after mutual gaze. *p < 0.05. *n* = 10. Error bars represent SEM. C) Schematic of human–dog interaction with (bottom) and without (top) petting. D) The interbrain activity couplings between frontal and parietal regions of dog and human increase after petting. *p < 0.05. *n* = 10. Error bars represent SEM. E) Interbrain correlation between frontal regions (top)/parietal region (bottom) of dog and human brain during mutual gaze + petting (black), mutual gaze alone (green), petting alone (orange), and mutual gaze + petting (theoretical) (gray). Kruskal–Wallis test. *p < 0.05. Error bars represent SEM.

To separate the effect of mutual gaze and petting on interbrain activity coupling, we designed the follow‐up tests: in control condition, the dog and the human interaction partner stayed in the same room without any social interactions. In interaction condition, the human interaction partner interacted with the dog by either mutual gaze only (**Figure** **2**A) or petting (30 g cm^−2^ petting pressure and 3–5 s per touch) only (Figure 2C). We observed that interbrain correlations in frontal and parietal regions dramatically increased from 0.010 ± 0.0048 and 0.013 ± 0.0054 without mutual gaze to 0.20 ± 0.0064 and 0.069 ± 0.0060 during mutual gaze, respectively (*p* < 0.05, Mann‒Whitley *U* test; Figure 2A,B). The correlations in frontal regions (0.20 ± 0.0064) were significantly higher than those in parietal regions (0.069 ± 0.0060) during mutual gaze (Figure 2B; *p* < 0.001, Mann‒Whitley *U* test).

Similarly, we found that the interbrain correlations in frontal and parietal regions significantly increased from 0.019 ± 0.0066 and 0.016 ± 0.0071 without petting to 0.090 ± 0.0071 and 0.17 ± 0.0096 during petting, respectively (*p* < 0.05, Mann‒Whitley *U* test) (Figure 2C,D); the interbrain correlations in parietal regions (0.17 ± 0.0096) were significantly higher than that in frontal regions (0.090 ± 0.0071) during petting (Figure 2D; *p* < 0.001, Mann‒Whitley *U* test).

It is plausible that a synergistic effect of both mutual gaze and petting may significantly enhancing interbrain activity coupling compared with the stimuli of mutual gaze or petting alone. To test this hypothesis, we compared the interbrain activity coupling during full social interactions (mutual gaze plus petting) with that during partial social interactions (mutual gaze or petting alone). The anaysis revealed that the interbrain correlation in the frontal and parietal regions of dogs and humans induced by mutual gaze or petting alone was significantly lower than that during full social interactions with both mutual gaze and petting (Figure 2E). More importantly, the simple sum of the effect of the two individual forms of stimuli was significantly lower than that of the full social interactions with both mutual gaze and petting (Figure 2E). These findings are consistent with a previous study showing that, compared with unimodal stimuli, multimodal stimuli induced a greater interbrain neural synchronization.^[^ ^24^ ^]^

### 2.3 A Leader–Follower relationship Emerges in Interbrain Neural Coupling of Interacting Humans and Dogs

To maintain cohesion in social species, all members of a group that act together must follow a set of basic rules, resulting in the emergence of a leader–follower relationship.^[^ ^21^, ^25^ ^]^ The leader–follower relationship is an important aspect of animal social life. It is essential for leaders to work toward building mutually beneficial partnerships with their followers and advancing the interests of the group as a whole.^[^ ^25^ ^]^ Previous studies in humans have found that interbrain activity coupling exhibits directionality when a leader–follower relationship is established in a social group.^[^ ^21^ ^]^ Specifically, leader emergence in a group discussion task was characterized by high neural synchronization between the leader and followers and that leadership could be predicted based on the directionality of interbrain activity coupling. Given domestic dogs are gregarious animals who live in a well‐established social hierarchy, they perceive their human owners as leaders.^[^ ^26^ ^]^

**Figure 3:**
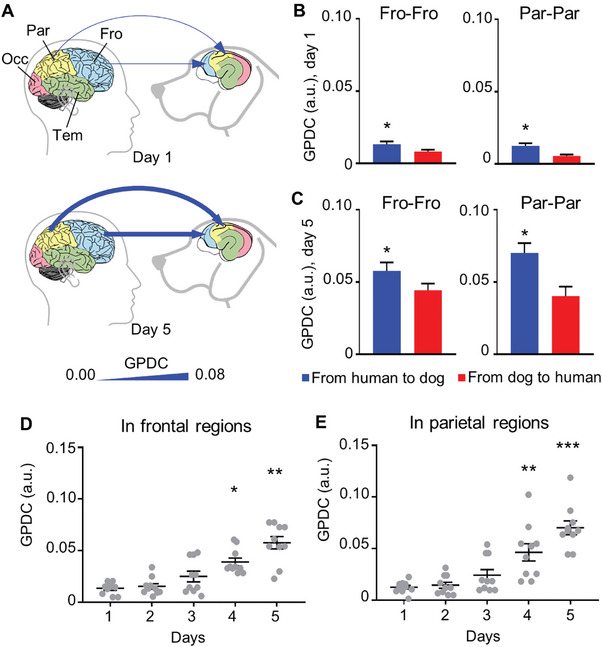
The direction of interbrain activity coupling is from human to dog and increases in five days with more social interactions. A) Brain regions involved in activity synchronization on the first and fifth day of social interactions and the direction of synchronization. The line width indicates the intensity of GPDC (generalized partial directed coherence). The blue arrow indicates direction of activity synchronization. B,C) The GPDC from human to dog (blue) and GPDC from dog to human (red) in frontal regions and parietal regions on the first and fifth day of social interactions. *p < 0.05 between the two directions, Friedman test. *n* = 10. Error bars represent SEM. D,E) The directionality of activity synchronization in frontal regions and parietal regions over five days. *p < 0.05, **p < 0.01, ***p < 0.001 compared with that on the first day, Friedman test.

To determine if the interbrain synchronization exhibited directionality during human–dog interactions, we used generalized partial directed coherence (GPDC) to measure the directionality of interbrain activity coupling. GPDC is a mathematical algorithm that uses the phase of cortical oscillations to determine the information flow, i.e., the directionality between the corresponding brain regions of socially interacting individuals.^[^ ^27^, ^28^ ^]^ The mean‐normalized power of the EEG data was used to compute GPDC values. We analyzed the EEG data of the brain regions involved in interbrain activity coupling across five days of social interactions. Interestingly, the direction of the synchronization was from human to dog throughout the 5‐d interaction periods (**Figure** **3**B,C), and there appeared to be a dramatic increase in GPDC with more repeated social interactions across five days (Figure 3A). We found that GPDC was significantly enhanced on the fourth and fifth days compared with the first day of social interactions in frontal regions and parietal regions (Figure 3D,E). We assumed that human's eye gaze and initiation of petting may drive the coupled brain activity. The linear regression results confirmed a significant positive relationship between social interaction time and GPDC (β = 0.011, SEM = 0.0014, *p* < 0.01 in frontal regions; β = 0.015, SEM = 0. 0019, *p* < 0.01 in parietal regions).

### 2.4 Disruption of Interbrain Neural Coupling between Humans and *Shank3* Mutant Dogs

*SHANK3* mutations are the most common and replicated genetic risk factors for ASD, a neuropsychiatric condition affecting ≈1% of the human population worldwide.^[^ ^29^, ^30^ ^]^ Persistent deficits in communication and social interaction across multiple contexts are core ASD symptoms.^[^ ^31^ ^]^ Children with severe ASD symptoms show a lower level of interbrain activity coupling with their parents during social interactions.^[^ ^32^ ^]^

Dogs are an effective and complementary animal model for studying social cognition and neuropsychiatric disorders, such as ASD in humans.^[^ ^33^, ^34^ ^]^ To address the challenges of rodent models (which have a limited translational value due to the differences in brain anatomy and behaviors from humans) and monkey models (which have a slow rate of reproduction and an extremely high cost), we generated and characterized an ASD model in dogs carrying heterozygous and homozygous *Shank3* mutations of −483+7, −496, and −1279+1 bp DNA indels in the coding region by the CRISPR/Cas9 genome editing technique; these mutant dogs showed clear autism‐like phenotypes as measured by a battery of behavioral assays such as the three‐chamber test and the human–dog interaction assays.^[^ ^34^, ^35^ ^]^

**Figure 4:**
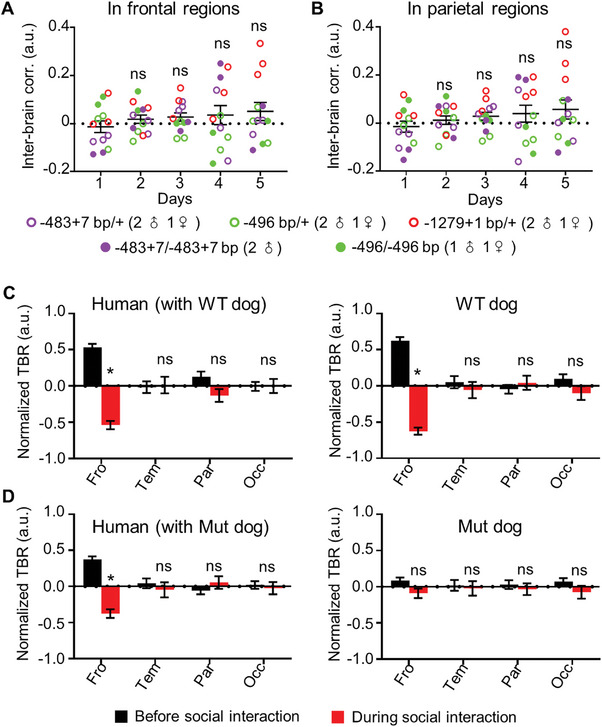
Loss of interbrain activity coupling and impaired attention in *Shank3* mutant dogs during human–dog interactions. A,B) Interbrain correlation in frontal‐frontal regions and parietal‐parietal regions during interactions between *Shank3* mutant and human over five days. ns: no significance, compared with the first day, Friedman test. C) The normalized theta/beta ratio (TBR) in each brain regions of human (left) and WT dogs (right) before (black) and during (red) full social interaction. *p < 0.05 in the frontal region. ns: no significance in other brain regions. *n* = 10. Error bars represent SEM. D) The normalized TBRs in each brain regions of human (left) and *Shank3* mutant dogs (right) before (black) and during (red) full social interaction. *p < 0.05. ns: no significance. *n* = 13. Error bars represent SEM.

To determine whether dogs carrying *Shank3* mutantions exhibited atypical interbrain activity coupling during interactions with humans, we performed a 5‐d human–dog interaction experiment on dogs carrying *Shank3* mutantions with the same protocol used for the wild‐type (WT) dogs. Unlike WT dog dyads reported in Figures 1 and 2, the two‐way ANOVA results showed no significant increase in the interbrain correlation of human–*Shank3* mutant dog dyads during various modes of social interactions (*p* > 0.05). Human–*Shank3* mutant dog dyads exhibited much lower interbrain correlation (0.023 ± 0.013 in the frontal regions and 0.024 ± 0.012 in the parietal regions) than human–dog (WT) dyads (0.22 ± 0.022 in the frontal regions and 0.21 ± 0.024 in the parietal regions) (*F*(1, 338) = 34.23, *p* < 0.001 in frontal regions and *F*(1, 339) = 32.14, *p* < 0.001 in the parietal regions) (**Figure** **4**A,B). The linear regression results further confirmed no significant relationship between social interaction time and interbrain correlation in the human–mutant dog dyads (β = 0.01, SEM = 0.01, *p* > 0.05 in frontal regions; β = 0.02, SEM = 0.01, *p* > 0.05 in parietal regions).

### 2.5 Reduced Joint Attention in Dogs Carrying *Shank3* Mutations during Interactions with Humans

The dogs carrying *Shank3* mutations showed a loss of interbrain activity coupling during interactions with humans in the frontal and parietal regions in the above tests. As the frontal and parietal regions are both associated with attention,^[^ ^36^ ^]^ we wondered whether there were attention deficits in *Shank3* mutant dogs during human–dog interactions. The ratio between the slow wave theta (4−8 Hz) and fast wave beta (13−30 Hz) band power, i.e., the theta/beta ratio (TBR), is a widely used EEG metric for assessing attention.^[^ ^37^ ^]^ By examining the TBR, one can gain insight into an individual's cognitive state and identify potential attention‐related abnormalities. A high TBR suggests a less attentive state, while a low TBR reflects a more attentive, focused state. Previous studies have shown that children with ASD and attention‐deficit/hyperactivity disorder (ADHD) had a significantly higher TBR than typically developing children in the resting state.^[^ ^37^, ^38^ ^]^ Similarly, children with ASD exhibit impaired attention to social cues by behavioral assays.^[^ ^39^, ^40^ ^]^

To determine whether dogs carrying *Shank3* mutantions exhibited impaired attention, we analyzed TBR in human–dog dyads during social interactions. The results showed that the TBR in the frontal but not other regions was significantly reduced in WT dogs but not in mutant dogs when engaging in social interactions with humans across five days (Figure 4C,D). These findings indicate impaired attention in dogs carrying *Shank3* mutantions.

### 2.6 A Single Dose of LSD Rescues Impaired Interbrain Coupling and Joint Attention in *Shank3* Mutant Dogs

Currently, there is a lack of effective pharmacological treatments specifically addressing the core phenotype of ASD: social deficits.^[^ ^41^ ^]^ Psychedelics, known for their hallucinogenic properties, are undergoing a renewed wave of scientific scrutiny, building on the pioneering research conducted in the mid‐twentieth century.^[^ ^42^ ^]^ Lysergic acid diethylamide (LSD) is one of these psychedelics. Recent studies in humans have shown that a single administration of LSD could enhance sociability, empathy and blood levels of oxytocin, a neuropeptide implicated in social behavior.^[^ ^43^, ^44^, ^45^ ^]^

**Figure 5:**
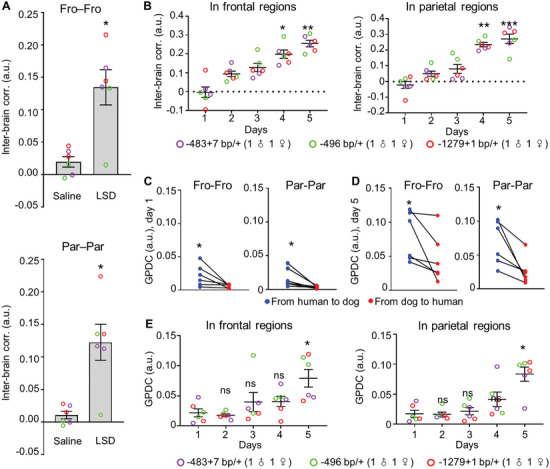
The effect of LSD on interbrain activity coupling. A) Interbrain correlation in frontal and parietal regions of *Shank3* mutant dog and human brain after saline and LSD treatment. *p < 0.05. *n* = 6. Error bars represent SEM. B) Interbrain correlation in frontal regions (left) and parietal regions (right) of socially interacting *Shank3* mutant dog after LSD treatment and human over five days. *p < 0.05, **p < 0.01, ***p < 0.001 compared with the first day, Friedman test. C,D) The GPDC from human to *Shank3* mutant dog (blue) and from *Shank3* mutant dog to human (red) in frontal regions and parietal regions on the first and fifth day of social interactions after LSD treatment. *p < 0.05. Friedman test. *n* = 6. Error bars represent SEM. E) GPDC representing the directionality of brain activity synchronization from human to dog in frontal regions and parietal region over five days after LSD treatment. *p < 0.05, ns: no significance compared with that on the first day, Friedman test.

To address if LSD had an effect on the brain activity coupling in the interacting dog–human dyads, we conducted a pilot study to determine an appropriate LSD dose at 7.5 µg kg^−1^ bodyweight, as 10 µg kg^−1^ bodyweight (inferred from previous reports on mice^[^ ^13^ ^]^) showed an apparent head‐shaking effect, while 5 µg LSD kg^−1^ bodyweight showed no recognizable effect on the behaviors. Previous studies showed that a single dose of LSD facilitates social reward learning for three weeks in mice^[^ ^13^ ^]^ and produces enduring therapeutic benefits for specific neuropsychiatric conditions.^[^ ^46^ ^]^ We therefore administered intramuscularly LSD at a single dose of 7.5 µg kg^−1^ bodyweight and examined its effect 24 h later. We found that human–*Shank3* mutant dog (after LSD treatment) dyads exhibited much higher interbrain correlation (0.13 ± 0.027 in the frontal regions and 0.12 ± 0.028 in the parietal regions) than human–*Shank3* mutant dog (after saline treatment) dyads (0.020 ± 0.008 in the frontal regions and 0.011 ± 0.006 in the parietal regions; *p* < 0.05) (**Figure** **5**A). The interbrain activity coupling significantly increased from −0.0030 ± 0.028 and −0.022 ± 0.022 on the first day to 0.26 ± 0.017 and 0.27 ± 0.030 on the fifth day in frontal regions and parietal regions, respectively (Figure 5B). The GPDC values from human to dog were higher than that from dog to human throughout the 5‐d interaction periods (Figure 5C,D). There was also an increase in GPDC from human to dog with repeated social interactions in five days; the GPDC significantly increased from 0.022 ± 0.0066 and 0.018 ± 0.0057 on the first day to 0.079 ± 0.015 and 0.084 ± 0.012 on the fifth day in frontal regions and parietal regions, respectively, after LSD treatment (Figure 5E).

**Figure 6:**
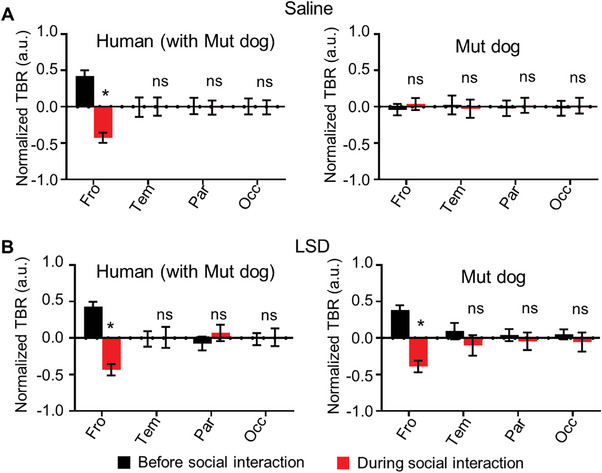
LSD rescued impaired attention in *Shank3* mutant dogs during human–dog interactions. A) The normalized theta/beta ratio (TBR) in each brain regions of human (left) and *Shank3* mutant dogs (right) before (black) and during (red) full social interactions after saline treatment. B) The normalized TBRs in each brain regions of human (left) and *Shank3* mutant dogs (right) before (black) and during (red) full social interactions after LSD treatment. *p < 0.05, ns: no significance in other brain regions; *n* = 6. Error bars represent SEM.

To determine whether LSD rescued impaired attention in dogs carrying *Shank3* mutantions, we analyzed TBR in human–*Shank3* mutant dog dyads and found that the TBR in the frontal regions but not in other brain regions was significantly reduced in *Shank3* mutant dogs after LSD treatment but not in saline‐treated mutant controls (**Figure** **6**A,B), indicating a rescue effect on attention by LSD in *Shank3* mutants.

## 3 Discussion

This study is the first to report and characterize interbrain activity coupling during cross‐species interactions. Our results show that the strength, direction, and attention‐associated brain regions of the interbrain activity coupling during human–dog interactions are similar to those during human–human interactions.^[^ ^9^, ^16^, ^17^ ^]^ Specifically, we discovered that the frontoparietal network is a critical brain network involved in interbrain activity coupling. The frontoparietal network is crucially involved in the attentional selection of sensory information.^[^ ^47^ ^]^ The distinct functions of the two brain regions, i.e., frontal for visual/facial cues and parietal for somatosensory cues,^[^ ^47^ ^]^ in the frontoparietal network may explain our findings that mutual gaze induced higher interbrain activity coupling in the frontal region, while petting induced higher interbrain activity coupling in the parietal region. A few studies have shown that the frontoparietal network plays a vital role in the pathophysiology of ASD.^[^ ^48^, ^49^ ^]^ Our findings suggest that dogs carrying *Shank3* mutantions exhibited impaired neural circuitry analogous to that in patients with ASD. By analyzing the TBR, which has been used as a biomarker of attention,^[^ ^37^ ^]^ we revealed impaired attention in dogs carrying *Shank3* mutations during human–dog interactions. Furthermore, previous studies have reported interbrain activity coupling in humans exhibits leader–follower directionality.^[^ ^21^, ^50^ ^]^ Our study extended these findings between humans to cross‐species human–dog dyads and indicated human‐to‐dog directionality of interbrain activity coupling during social interactions.

The brain activity coupling is disrupted in socially interacting human‐mutant dog dyads. The reason for this is not known at the moment, but it may be caused by reduced neural responsiveness in the mutant dogs. This notion is supported by the fact that light stimulation induced slower and reduced pupil constriction in *Shank3* mutant dogs,^[^ ^35^ ^]^ indicating impaired neural signal processing efficiency.

Previous studies demonstrate that LSD enhances social behavior in mice,^[^ ^51^, ^52^ ^]^ suggesting a therapeutic potential of LSD for social deficts in ASD patients. Nardou et al. recently demonstrated in mice that the ability to reopen the critical period of social reward learning is a shared property across psychedelic drugs, including LSD.^[^ ^13^ ^]^ We showed for the first time that a single dose of LSD rescued impaired interbrain coupling and joint attention in *Shank3* mutant dogs, suggesting that LSD may potentially ameliorate social deficits in ASD, though the mechanism underlying the rescuing effect remains unclear.

Nevertheless, there are still a few limitations of the present study. Given the large size and thickness of the temporalis muscle in dogs, extraction of high‐quanlity EEG signals from the temporal region of the dog brain was not feasible. Therefore, we cannot rule out the possibility of interbrain activity coupling in the temporal regions. Large physical movements generate substantial motion artifacts in EEG data, thereby preventing us from utilizing more natural settings of human–dog interactions, such as free‐moving play for studying interbrain activity coupling. We also note that in the absence of source reconstructions, we cannot firmly conclude that the brain activity coupling based on 16‐channel scalp EEG analysis occurrs exclusively in fronto‐parietal brain regions.

Despite these limitations, the present study provides direct evidence that human–dog social interactions involve coupled neural activities, and joint attention contributes to the interbrain activity coupling between dogs and humans. In addition, mutations of the high‐risk autism gene *Shank3* in dogs lead to attention deficits. Moreover, the social deficits of *Shank3* mutant dogs were rescued by a single dose of LSD. We believe the unique experimental paradigm and the methodologies developed in the present study are useful for elucidating the neural mechanisms underlying social deficits in ASD. Furthermore, our findings suggest potential interbrain activity biomarkers for ASD diagnosis and development of engineered non‐hallucinogenic analogs of LSD to correct social deficits. Further studies on brain activity coupling could deepen our understanding of the neural mechanisms underlying the social interactions among typically developing individual humans and the social deficits in patients with psychiatric disorders, including ASD.

## 4 Experimental Section

### Animals

All animal‐related protocols were approved in advance by the Animal Care and Use Committee of the Institute of Genetics and Developmental Biology (AP2019037). Ten control Beagle dogs (1–2 years old, all males; provided by Beijing Sinogene Biotechnology Co. Ltd.), nine heterozygous *Shank3* mutants (−1279+1 bp/+, −496 bp/+, and −483+7 bp/+, *n* = 3 for each genotype, including two males and one female per genotype, 1–2 years old), and four homozygous *Shank3* mutants (−496/−496 and −483+7/−483+7 bp, *n* = 2 for each genotype, including one male and one female per genotype, 1–2 years old) previously generated and characterized^[^ ^34^ ^]^ were used for various experiments in the present study. The dogs were housed in pairs after weaning at postnatal day 50 in 2 m × 0.9 m × 1.5 m (length × width × height) cages and maintained on a 12/12 h dark‐light cycle, with a humidity of 40%–60% and a temperature of 22–24 °C. During non‐experimental periods, dogs were exposed to the breeders only. The dogs were fed with Royal Canine Chow (Royal Pet Food Company Ltd., France) twice daily from 08:00–10:00 and 15:30–17:00. Based on regular veterinary assessments, all dogs were in good health at the time of experiments. All tests were performed at the timeslot of 9:00–12:00 or 14:30–17:00, and none of the animals had any prior contact with the experimenters.

### Behavioral Experiments

Prior to EEG data acquisition, the dogs were trained over three months, approximately three times a week, for the EEG task. Since muscle movements cause artifacts in EEG data, the dogs were trained with a positive operant conditioning method (clicker) to sit still.

Before the experiment, each dog was guided into an area (2 × 3 m) surrounded by fences, and allowed to explore freely for 10 min. For the test of no interaction in separate rooms, the dog and the experimenter stayed in separate rooms. Subsequently, an individual issued a command for the dog to sit calmly and exited the room prior to the initiation of the EEG recording. The EEG was then recorded for a duration of five minutes. During EEG recordings, the dog and the experimenter were in the same room for a 5‐min period when no interactions (mutual gaze or petting) took place. Conversely, when EEG recordings were taken during human–dog interactions, the experimenter commanded the dog to sit down quietly, then the dog and the experimenter were in the same room and engaged in a five‐minute interaction, which involved the experimenter petting the dog's neck or back at a frequency of once every 3–5 s, and mutual gaze (continuously looked at the dog). An observer recorded the duration of mutual gaze between the experimenter and the dog using a camera. Records with a total mutual‐gaze duration of less than 2 min were excluded. Each experiment was initiated by the human in social interactions. Every dog completed one session of the assay per day, for five consecutive days.

An assay was designed for investigating the effect of mutual gaze and petting on the interbrain correlation between dogs and humans. Before conducting the petting assay, the effect of different petting parameters were tested: petting pressures from 20 to 40 g cm^−2^ and petting frequencies from 1–3 to 5–7 s per touch. There were no significant differences in the interbrain activity correlation at different petting parameters (Figure S4, Supporting Information). 30 g cm^−2^ petting pressure and 3–5 s per touch were therefore used in the following assay. For no mutual gaze/petting control (first stage), the dog and the familiar experimenter stayed in the same room without petting and mutual gaze (the experimenter avoided to look at the dog to rule out the effect of visual stimulation by eye contact). For mutual gaze/petting (second stage), the experimenter interacted with the dog only by mutual gaze/petting. The “first stage” and “the second stage” sequences were performed for each dog on the same day, at an interval of 10 min, one time per animal per day. For mutual gaze, the experimenter commanded the dog to sit down quietly. An observer recorded the duration of mutual gaze between the experimenter and the dog using a camera. Records with a total duration of less than 2 min were excluded. To rule out the effect of stage orders, the order of the stages was reversed, and the process was repeated. Only one person, who understood the research objectives, interacted with dogs throughout the whole study.

### Electrophysiological Recording

The brain activity of both partners was simultaneously recorded with a dual‐EEG recording system using a 32‐channel wireless EEG amplifier manufactured by Bio‐Signal Technologies, which performs at a sampling rate of 500 Hz, and stores the digitized data on an onboard SD card. The system was equipped with helmets for both humans and dogs with 16 silver/silver chloride coated EEG electrodes (Greentek, China), according to the international 10/20 system and connected to two synchronized amplifiers to guarantee millisecond‐range synchrony between the EEG recordings from both dog and human (Figure S1, Supporting Information).^[^ ^53^ ^]^ To attach the electrodes to the skin, the hair from the top of the dog's head was shaved, and the skin was applied with medical conductive gel (Greentek, China). Drops of tissue glue (3M, USA) were applied on the corners of the electrode pads to enhance the attachment of electrodes to the skin. In addition, medical elastic tape (3M, USA) was applied on top of the electrodes to ensure their close attachment.^[^ ^54^ ^]^ The impedances were maintained below 10 kΩ.

### EEG Data Collection and Preprocessing

The preprocessing was conducted using Matlab (The MathWorks) with Fieldtrip toolbox.^[^ ^55^ ^]^ To obtain scalp EEG, the raw voltage traces were first low‐pass filtered using a tenth‐order Butterworth filter with a cut‐off frequency of 250 Hz. By visual inspection, artifacts were observed in EEG recording, in the form of large amplitude (e.g., >300 uv), transient, irregular voltage fluctuations that were visually distinct from the normal EEG signal.^[^ ^11^ ^]^ The artifacts were manually removed, and then the linear trends were removed. When analyzing EEG signal, after removing an artifact from an EEG trace, the resulting gap was closed by joining the two ends of the trace.^[^ ^14^ ^]^

For each EEG trace, its spectrogram was calculated as follows. Power spectra were calculated for 1 s sliding windows of the EEG signal, with no overlap between consecutive windows. Delta waves (0–4 Hz) in dogs and humans are generally associated with deep sleep or cerebral lesions, and gamma waves (>30 Hz) are associated with complex cognitive processing. Hence, the 4–30 Hz frequency band was selected, which encompasses theta, alpha, and beta waves, as the range for analysis. The power spectra were computed at integer frequencies from 4 to 30 Hz, using the multitaper method with a time half bandwidth product of 4. To analyze and visualize different frequencies on equal footing, for each EEG spectrogram, the power at each frequency was separately peak‐normalized, i.e., power at each frequency was divided by the peak power at that frequency (Figure S5, Supporting Information).^[^ ^11^ ^]^

Artifact identification in the EEG data was conducted through a systematic, multistep process (Figure S5, Supporting Information). Initial visual inspection of the EEG data was carried out, both at a global level and on a channel‐by‐channel basis, to identify obvious anomalies indicative of potential artifacts. This was followed by a more detailed examination focusing on the characteristic features of various artifact types. Ocular artifacts, originating from eye movements and blinks, were identified based on their large potential shifts, particularly evident in the frontal electrodes due to their proximity to the eyes. Eye blinks were distinguished by a characteristic positive deflection followed by a negative trough. Eye movements, on the other hand, caused shifts in the baseline of the EEG signal. Muscle artifacts or electromyographic (EMG) noise were recognized by their high‐frequency content, often masking the underlying EEG signal. These artifacts were particularly noticeable in the temporal and frontal electrodes, which are positioned close to the facial and neck muscles. Cardiac artifacts, reflecting the electrical activity of the heart, were recognized by their rhythmic, sharp deflections. These artifacts were most clearly observed in electrodes positioned near the neck, where the electrical activity of the heart can propagate most easily. Environmental artifacts, including electrical noise from power lines or equipment, were identified based on a characteristic 50 or 60 Hz oscillation, depending on the local power standards. Electrode artifacts, arising from issues such as poor scalp–electrode contact, broken wires, or disparate impedance between electrodes, were identified based on abrupt large deflections, flat signals, or “noisy” signals. Channels showing a significantly different signal compared with others were scrutinized for potential electrode artifacts.

Wavelet transformation was utilized as a preliminary step for the decomposition of EEG signals into different frequency bands.^[^ ^14^ ^]^ This mathematical technique involves the convolution of the EEG signal with wavelets, which are adept at capturing both time and frequency information. The wavelet transformation effectively isolates the frequency components that are characteristic of brain activity, as well as those that are indicative of artifacts (Figure S5, Supporting Information).

Following the wavelet transformation, the decomposed signals were subjected to analysis by a neural network.^[^ ^15^ ^]^ The neural network used in the study was an artificial neural network (ANN) comprising multiple layers of interconnected nodes. The ANN was trained on a dataset of EEG signals with known artifacts, enabling it to learn the distinguishing features of brain activity and artifacts within the frequency domain. The weights of the connections between the nodes were iteratively adjusted during the training phase to minimize the classification error. The integration of wavelet transformation and neural networks was capable of discerning between genuine EEG signals and artifacts (Figure S5, Supporting Information). Finally, about 14.8% of the raw data were removed by visual examinations and the integrated approach.

### Drug Preparation and Administration

LSD was administered intramuscularly at a single dose of 7.5 µg kg^−1^ bodyweight to each test dog. USP‐grade saline (0.9% NaCl) was used as vehicle control. Only dogs were given LSD in the interacting dyads.

### Calculation of Pearson Correlation Coefficient and GPDC

To calculate the Pearson correlation coefficient between two vectors x and y, which were mean‐normalized power of EEG in this study, the means of both vectors were first calculated, then these means were used to standardize each value in the vectors by subtracting the mean and dividing by the standard deviation. The resulting standardized values were multiplied together and summed up to give a numerator, while the standard deviations of the original vectors were squared and multiplied together to give a denominator. Finally, the numerator was divided by the square root of the denominator to get the Pearson correlation coefficient, which measures the linear relationship between the two vectors.^[^ ^56^ ^]^

GPDC is a measure that can reveal causal influences between multiple time series within a multivariate system.^[^ ^28^ ^]^ The basic idea is to compute the coherence between each pair of time series, and then remove the effects of all other time series to isolate the direct causal influence between the two time series of interest.

To compute GPDC, preprocessed data in the form of a matrix X that has multiple channels or time series were taken. The frequency range of interest was first defined. Then, a multivariate autoregressive (MVAR) model was used to obtain the coefficient matrix A and noise covariance matrix SIG.

To obtain the power spectral density matrix S from A and SIG, the transfer function matrix H was first calculated by taking the inverse Fourier transform of the coefficient matrix A for each frequency point. It then multiplied H with its Hermitian transpose, conjugating its off‐diagonal elements, and added SIG to obtain an estimate of the power spectral density matrix S.

The coherence matrix C was computed by taking the absolute squared value of the normalized cross‐spectral density between each pair of time series. Specifically, for each frequency point, the cross‐spectral density between two time series was divided by the product of their individual power spectral densities.

Next, the generalized partial directed coherence matrix G was computed by removing the effects of all other time series from the coherence between each pair of time series of interest. Specifically, for each frequency point, the coherence between two time series was taken and multiplied it by the complex conjugate of the coefficient between the other time series and the receiving time series. It was then normalized by dividing it by the square root of the product of the two time series’ individual power spectral densities and a correction factor that depends on the other time series. Finally, the GPDC values in the frequency range of interest were extracted.

### Statistic Analysis

Statistical analysis was performed using GraphPad Prism (www.graphpad.com). Differences between interbrain correlation without social interactions in separate rooms, without social interactions in the same room, and with social interactions in the same room were analyzed by two‐tailed Friedman test. Differences between interbrain correlation/GPDC over five days of social interactions were analyzed by two‐tailed Friedman test. Differences between within‐trial and between‐trial correlation were analyzed by two‐tailed Mann–Whitney *U* test. Differences between interbrain correlation with and without mutual gaze/petting were analyzed by two‐tailed Mann–Whitney *U* test. Differences between interbrain correlation of wild‐type dogs and *Shank3* mutants were analyzed by two‐way ANOVA. Differences between theta/beta ratio with and without social interactions were analyzed by multiple Mann–Whitney *U* test. Differences between interbrain correlation during full social interactions (mutual gaze and petting) and interbrain correlation during partial social interactions (mutual gaze or petting) were analyzed by one‐way ANOVA. Significance was set to be *p* < 0.05, adjusted for multiple comparisons by the Holm–Šídák method.

### Ethical Statement

All animal‐related protocols were approved in advance by the Institutional Animal Care and Use Committee of the Institute of Genetics and Developmental Biology (AP2019037).

## Conflict of Interest

The authors declare no conflict of interest.

## Author Contributions

W.R., S.Y., K.G., C.L., and Y.Q.Z. conceptualized the study. W.R. developed tools and methods for the study.  W.R. conducted the experiments and analyzed the data. All authors discussed the results. Y.Q.Z. acquired funding. Y.Q.Z. administrated the project. Y.Q.Z., C.L., K.G., and S.Y. supervised the study. W.R. wrote the original draft. Y.Q.Z., C.L., K.G., and S.Y. reviewed and edited the final report.

## Supporting information

Supporting Information

## Data Availability

The data that support the findings of this study are available from the corresponding author upon reasonable request.
