# Supplementary material for: Disrupted Human–Dog Interbrain Neural Coupling in Autism‐Associated Shank3 Mutant Dogs
Source: Adv Sci (Weinh). 2024 Sep 11;11(41):2402493. doi: 10.1002/advs.202402493 (PMC11538694; doi:10.1002/advs.202402493)
Supplement: Supplementary file 1 — Supporting Information [file ADVS-11-2402493-s001.docx]

Supporting Information


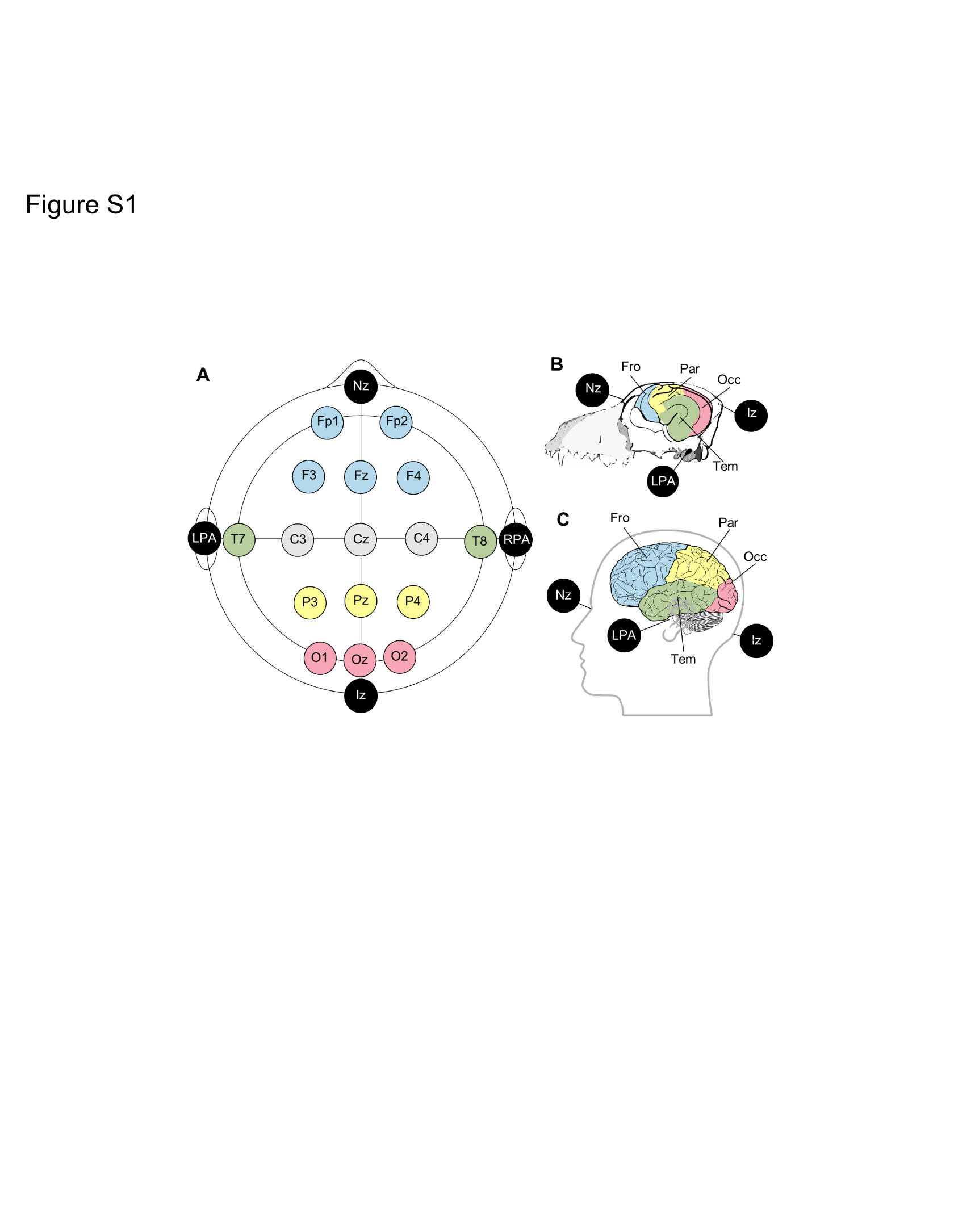


**Figure S1. Scalp electrode locations for simultaneous wireless EEG recording in human and dog. (A)** Location of 16 scalp EEG electrodes (blue: frontal region; gray: central region; yellow: parietal region; green: temporal region). Nz: the bridge of the nose; LPA: the left ear canals; RPA: the right ear canals; Iz: the little bump at the very back of the skull. **(B)** Division of dog brain regions (blue: frontal region; yellow: parietal region; green: temporal region; red: occipital region). **(C)** Division of human brain regions (blue: frontal region; yellow: parietal region; green: temporal region; red: occipital region).


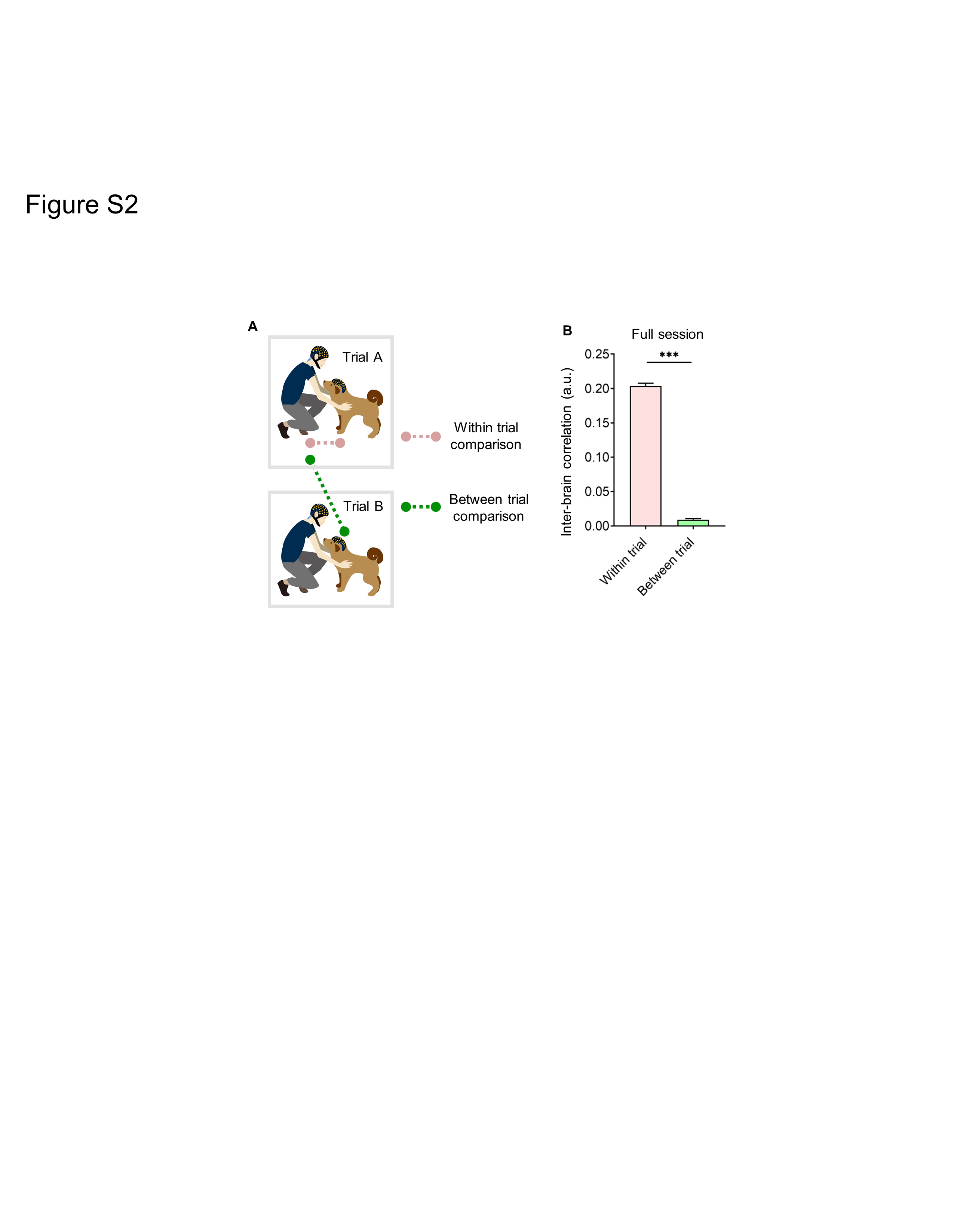


**Figure S2. Interbrain correlations of human–dog dyads from different trials. (A)** Schematic diagram of the analysis of interbrain correlation within the same trial and between different trials. **(B)** Comparison of interbrain correlation within the same trial and between different trials. ****p* < 0.001. Error bars represent SEM.


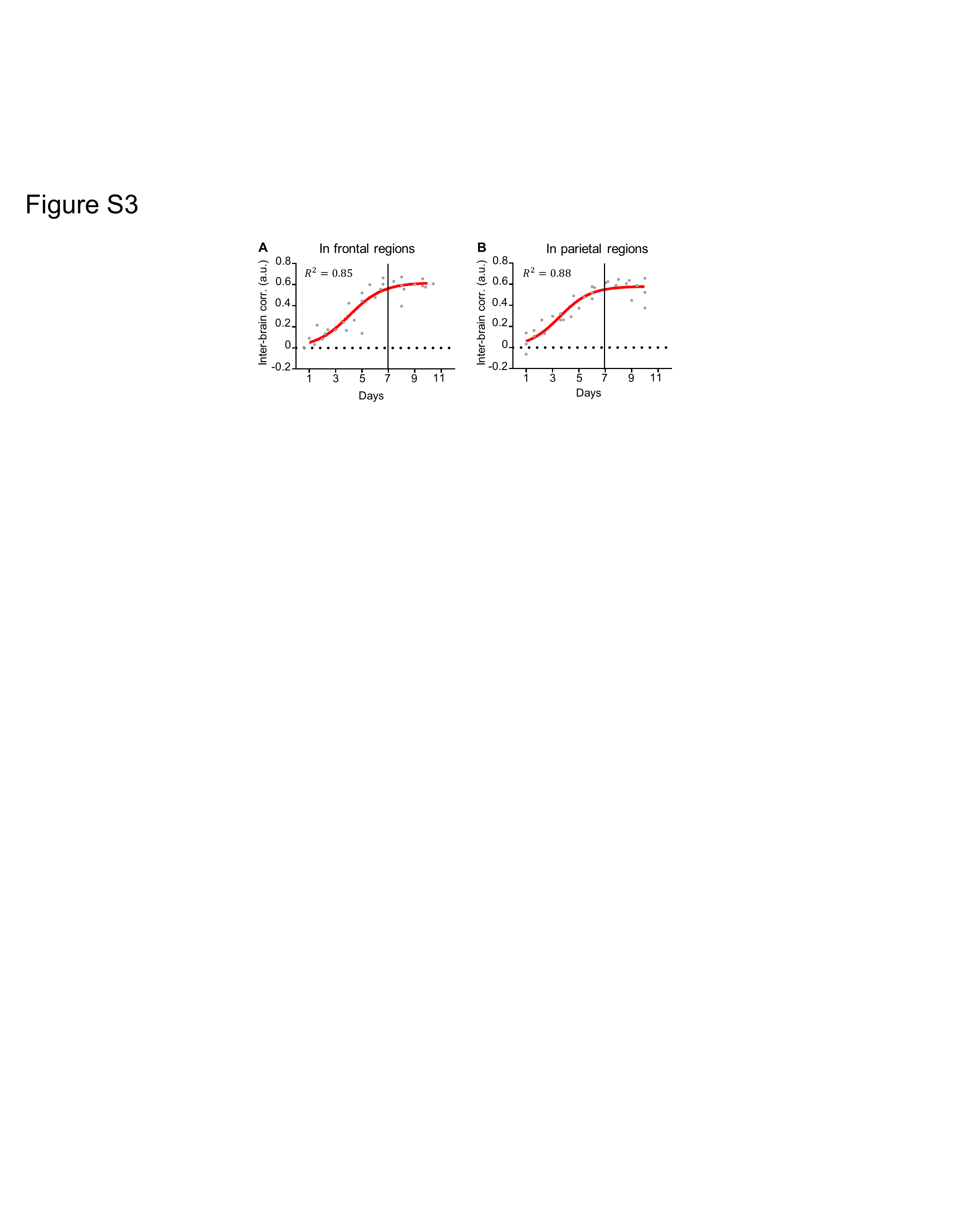


**Figure S3. Interbrain activity coupling reached a plateau after 8 days of social interactions. (A)** Estimated regression curve obtained by means of a logistic growth curve model linking social interaction time to interbrain correlation (between dog and human frontal region). **(B)** Estimated regression curve obtained by means of a logistic growth curve model linking social interaction time to interbrain correlation (between dog and human parietal region). n = 3. *R^2^* represents the correlation coefficient of the linear regression.


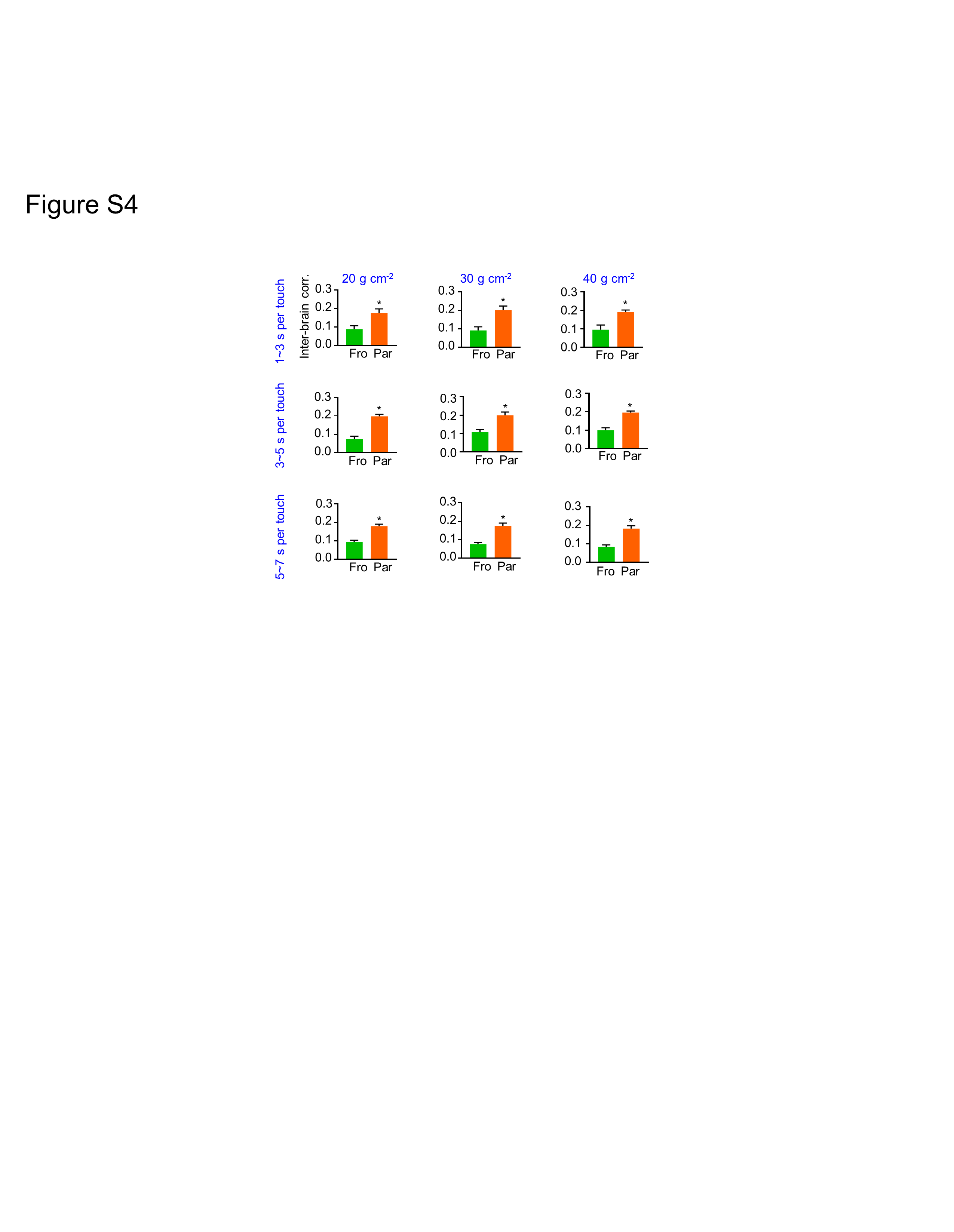


**Figure S4. The effect of different petting parameters on interbrain activity coupling.** The interbrain activity couplings between frontal and parietal regions of dog and human, with petting pressures ranging from 20 g cm^-2^ to 40 g cm^-2^ and petting frequencies ranging from 1~3 s to 5~7 s per touch. **p* < 0.05. n = 3. Error bars represent SEM.


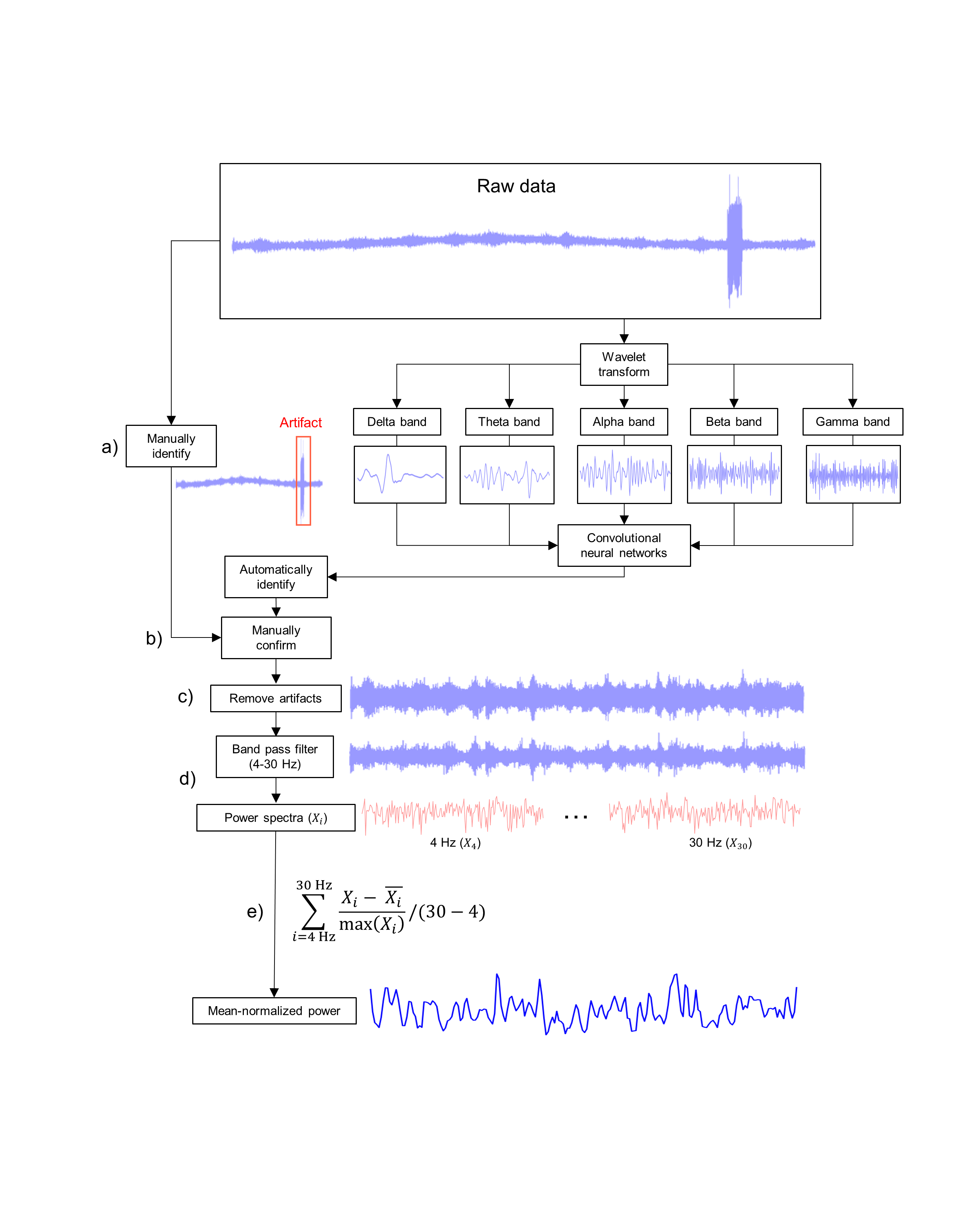


**Figure S5. The processing pipeline for EEG raw data.**

The EEG raw data were processed in the following steps: a) manually identifying the artifacts and feeding the data features extracted by wavelet transform into a convolutional neural network (CNN); b) manually confirming artifacts assisted by CNN; c) removing artifacts; d) computing power spectrum at integer frequencies from 4 to 30 Hz; e) peak-normalizing the power traces subtracted by the mean power at each frequency and then averaging the power traces.
